# T-Helper Cell Subset Response Is a Determining Factor in COVID-19 Progression

**DOI:** 10.3389/fcimb.2021.624483

**Published:** 2021-02-26

**Authors:** Francisco Javier Gil-Etayo, Patricia Suàrez-Fernández, Oscar Cabrera-Marante, Daniel Arroyo, Sara Garcinuño, Laura Naranjo, Daniel E. Pleguezuelo, Luis M. Allende, Esther Mancebo, Antonio Lalueza, Raquel Díaz-Simón, Estela Paz-Artal, Antonio Serrano

**Affiliations:** ^1^ Department of Immunology, Hospital Universitario 12 de Octubre, Madrid, Spain; ^2^ Departamento de Inmunologá, Instituto de Investigación, Sanitaria Hospital 12 de Octubre (imas12), Madrid, Spain; ^3^ Department of Internal Medicine, Hospital Universitario 12 de Octubre, Madrid, Spain; ^4^ Departamento de Medicina, Facultad de Medicina, Universidad Complutense de Madrid, Madrid, Spain; ^5^ Departamento de Inmunología, Oftalmología y Otorrinolaringología, Facultad de Medicina, Universidad Complutense de Madrid, Madrid, Spain; ^6^ Department of Epidemiology, Biomedical Research Centre Network for Epidemiology and Public Health (CIBERESP), Madrid, Spain

**Keywords:** COVID-19, SARS-Cov2, T-helper, immunity, cytokines

## Abstract

The immune response type organized against viral infection is determinant in the prognosis of some infections. This work has aimed to study Th polarization in acute COVID-19 and its possible association with the outcome through an observational prospective study. Fifty-eight COVID-19 patients were recruited in the Medicine Department of the hospital “12 de Octubre,” 55 patients remaining after losses to follow-up. Four groups were established according to maximum degree of disease progression. T-helper cell percentages and phenotypes, analyzed by flow cytometer, and serum cytokines levels, analyzed by Luminex, were evaluated when the microbiological diagnosis (acute phase) of the disease was obtained. Our study found a significant reduction of %Th1 and %Th17 cells with higher activated %Th2 cells in the COVID-19 patients compared with reference population. A higher percent of senescent Th2 cells was found in the patients who died than in those who survived. Senescent Th2 cell percentage was an independent risk factor for death (OR: 13.88) accompanied by the numbers of total lymphocytes (OR: 0.15) with an AUC of 0.879. COVID-19 patients showed a profile of pro-inflammatory serum cytokines compared to controls, with higher levels of IL-2, IL-6, IL-15, and IP-10. IL-10 and IL-13 were also elevated in patients compared to controls. Patients who did not survive presented significantly higher levels of IL-15 than those who recovered. No significant differences were observed according to disease progression groups. The study has shown that increased levels of IL-15 and a high Th2 response are associated with a fatal outcome of the disease.

## Introduction

Coronavirus disease 2019 (COVID-19) is an emergent condition caused by SARS-Cov2 infection that manifests with a wide spectrum of clinical profiles (Diao et al., 2020; Qin et al., 2020; Wang et al., 2020) and that presents a medical challenge due to its contagiousness rates (Guo et al., 2020; Rothan and Byrareddy, 2020). In general, COVID-19 begins as a mild disease with fever, cough, vomiting, myalgia, fatigue or diarrhea similar to those symptoms produced by a seasonally circulating common cold coronavirus. In many cases, the symptoms are so weak, even null, that the condition is not observed (Gao et al., 2020; Kim et al., 2020). However, some COVID-19 patients present lung involvement with severe pneumonia, pulmonary edema, acute distress respiratory syndrome (ARDS) and even death (2.5–7.2% of symptomatic patients) (Guan et al., 2020; Rothan and Byrareddy, 2020; Vardhana and Wolchok, 2020; Yang et al., 2020; Yuki et al., 2020). The disease severity has been associated with ageing and cardiovascular comorbidities (Huang W. et al., 2020; Hurwitz, 2020).

Among others parameters, mild and severe forms of the COVID-19 are characterized by a marked decrease in the total number of peripheral blood lymphocytes including T cells, both CD4+ and CD8+, B lymphocytes and Natural Killer (NK) cell (Song et al., 2020). It is interesting to note that T cells from severe patients present an effector, activated and senescent phenotype mainly associated with CD8+ cells (Vardhana and Wolchok, 2020). Furthermore, cytokine storm is the most life-threatening complication associated with COVID-19 (Schett et al., 2020; Buszko et al., 2020), characterized by a hyperactivated and pro-inflammatory state of the immune system. These features highlight the importance of the immune system response in the physiopathology of the disease (Chen and Wherry, 2020; Diao et al., 2020; Huang C. et al., 2020; Tan et al., 2020; Weiskopf et al., 2020; Vabret et al., 2020; Xu et al., 2020; Zheng et al., 2020; Zheng et al., 2020; Zhou et al., n.d).

The adaptive immune system, especially T cells, plays an important role against viral infections (Diao et al., 2020). T cell responses can mainly be polarized into effector cytotoxic cells (CTLs) and T helper (Th) cells (Mescher et al., 2006; Zhu et al., 2010). Th cells are subdivided into Th1, Th2, Th9, Th17 and follicular helper T cells (Tfh) subsets, each one having a characteristic function against infections (Alberts et al., 2002; Wan, 2010). Two major subtypes of Th cells coordinate responses to infection. One of them, the Th1 subset, coordinates the cell-mediated response, which is essential in macrophage, CTLs and NK activation *via* IL-2 and INF-gamma. Another one, the Th2 cells, which coordinates the humoral response, activates eosinophils, basophils and mast cells *via* IL4 and IL-6. In some infections, the type of Th lymphocytes that coordinates the immune response can condition the evolution of the clinical process. The polarization of the Th response will be determinant in the outcome of the disease (Mosmann and Sad, 1996; Infante-Duarte, 1999).

This phenomenon is well known in viral diseases such as lymphocytic choriomeningitis, where robust Th1 response is associated with a good prognosis (asymptomatic), whereas Th2 responses lead to anorexia and Wasting Syndrome (persistent viral load) (Kamperschroer et al., 2020). A similar situation is also observed in Human Immunodeficiency Virus (HIV) infection. Patients infected with HIV, who show a Th1 response, are seronegative and do not develop acquired immunodeficiency syndrome (AIDS), whereas Th2 patients become seropositive and evolve to AIDS (Clerici and Shearer, 1993; Mosmann and Sad, 1996; Calarota and Weiner, 2004).

The Th1/Th2 balance in COVID-19 has been associated with the outcome of the disease. When the viral infection is established, an appropriate immune response by Th1 is able to clear it discreetly. However, if this immune response is not well organized, an exacerbated reaction precedes the cytokine storm, priming Th2 cells that are linked to poor prognosis (Neidleman et al., 2020; Roncati et al., 2020). Thus, Th polarization seems to play an important role in determining COVID-19 severity, although it is still not totally understood.

This study has aimed to study the percentage and activation grade of Th subsets as well as the cytokine production in the acute moment of the COVID-19 and its association with outcomes.

## Materials and Methods

### Study Design

A prospective observational study that enrolled patients in the initial stages of COVID-19 in a Spanish tertiary university hospital was conducted. Peripheral blood lymphocyte immunophenotypes and cytokines levels were evaluated at the time of diagnosis. Patients were followed-up until discharge or death.

### Patients

A random sample of 58 patients with suspicion of COVID-19 were sequentially enrolled in the emergency department of the Hospital Universitario 12 de Octubre (Madrid, Spain) during 2020. Inclusion criteria were 1) Adult patients (>18 years) with symptoms of COVID-19 infection 2) Clinical characteristics or radiological findings that required hospital admission 3) Confirmed diagnosis by RT-PCR or serologic test at any moment of the disease.

As three subjects were lost to the follow-up, finally, 55 patients were included in the study. A reference group of 21 anonymous healthy blood donors representing the general population was established to compare the Th subset.

In tandem, 15 patients with symptoms consistent with COVID-19 initial infection (fever, headache, and cough), but who were SARS-Cov2 RT-PCR negative were recruited in order to compare peripheral blood lymphocyte immunophenotypes with confirmed COVID-19 patients. This group was named “COVID-Like.” All the patients in this group had a benign evolution and none required admission to the Intensive Care Unit (ICU).

A new cytokine anonymous control group (obtained before the pandemic) was created with 94 blood donors (other than those in the reference group) in order to compare cytokine levels with the COVID-19 cohort.

**Figure 1:**
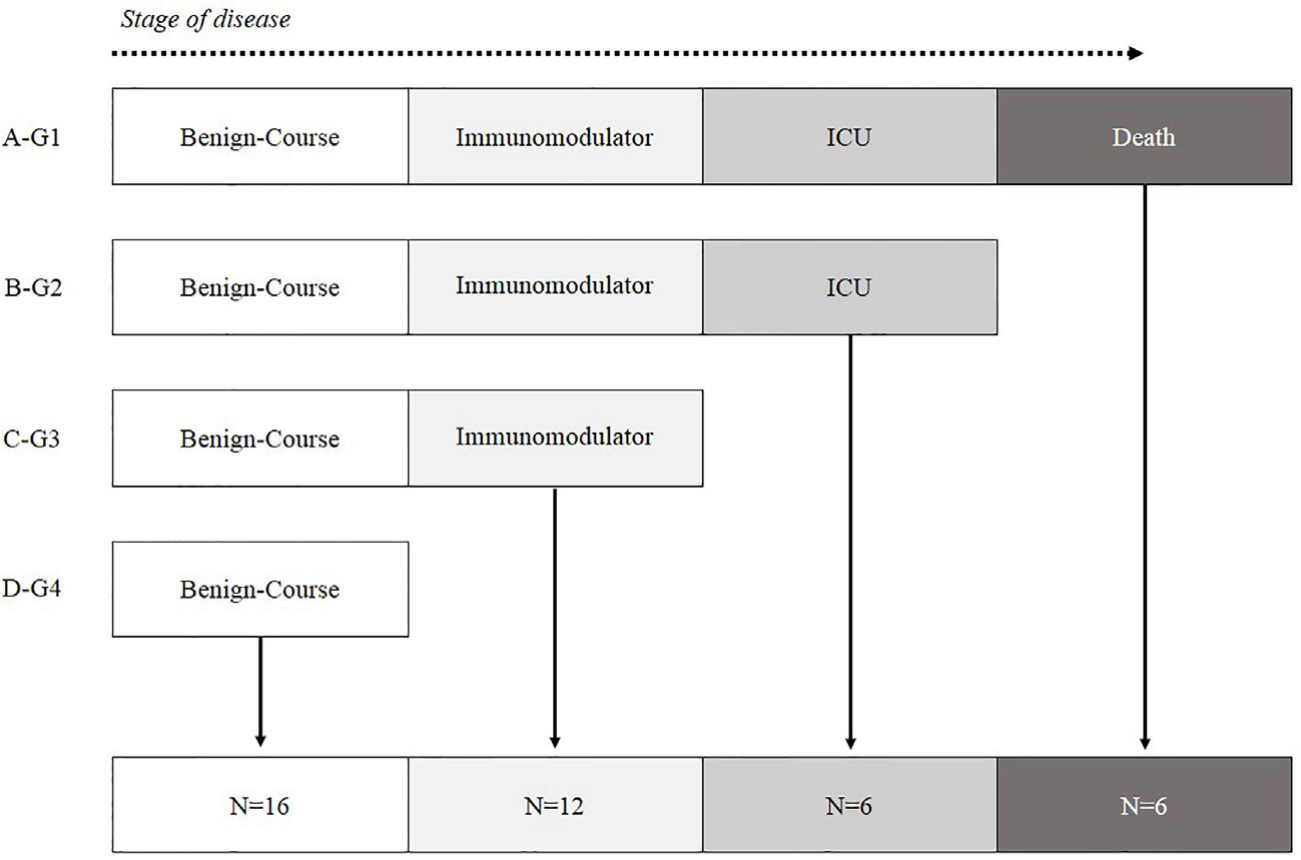
Patient classification according to the most critical event during hospitalization. **(A)** G1-Hospitalized patients who died. **(B)** G2-Alive patients who ended up in ICU. **(C)** G3-Hospitalized patients with poor prognosis characteristics who needed immunomodulatory treatment. **(D)** G4-Hospitalized patients with mild symptoms who did not need immunomodulatory treatment.

The remaining 55 patients recruited in this study were divided into four groups according to their evolution and most critical event 1) Patients who died (Group-1, Death, N = 9), 2) Patients who were treated in ICU (Group-2, ICU, N = 7), 3) Patients with severe involvement who required immunomodulatory treatment (Group-3, Immunomodulators, N = 14) and 4) Patients with a benign course that did not require complex therapies (Group-4, Benign Course, N = 25) (**Figure 1**).

Patients were also classified according to the worst SpO_2_/FiO_2_ (blood oxygen saturation/fractional inspired oxygen) during their disease into 1) Ventilatory failure, SpO_2_/FiO2<300. 2) Normal ventilation, SpO2/FiO2>300 (No ventilatory failure).

Diagnosis was made by RT-PCR in 73% of the patients using nasopharyngeal or oropharyngeal swabs as well as sputum samples. For those patients with a negative RT-PCR, the serologic diagnostic test against the spike and nucleocapsid protein was performed (27%) (Anti-SARS-Cov-2 ELISA (IgG/A) and Anti-NCP-SARS-Cov-2, (IgM/IgG) Euroimmun, Germany 2020, respectively).

### Ethics Statement

This study was conducted in accordance with the principles of the Declaration of Helsinki and was approved by the Clinical Research Ethics Committee of the University Hospital 12 de Octubre (reference no. 20/167). Oral or written informed consent was obtained from all patients.

### Study Definitions

COVID-19 case was defined as a person fulfilling both the clinical and laboratory criteria of COVID-19.
COVID-19 laboratory criteria were a positive result for severe acute respiratory syndrome coronavirus 2 (SARS-CoV-2) according to reverse transcription polymerase chain reaction (RT-PCR) or serological assays, performed by nasal swab sampling and sera or plasma, respectively.

### Data Collection

An anonymized database containing the patient data, including demographic, clinical and laboratory data from the electronic medical record, was created. Laboratory parameters included D-dimer, lactate dehydrogenase (LDH), C reactive protein (CRP), ferritin and the number of lymphocytes (lymphopenia was defined as total lymphocyte count of less than 0.85 × 10^9^/liter).

### Samples

Sera and EDTA-treated blood samples were collected in the first 24 h after hospitalization with a median of 7 days from the beginning of the symptoms before treatment.

### Flow Cytometry

Peripheral blood mononuclear cells (PBMCs) were isolated from EDTA-treated whole blood samples by density gradient centrifugation using Lymphoprep ™ (Fresenius Kabi, Oslo, Norway).

Peripheral blood lymphocytes (PBL) were isolated and stained with the following fluorochrome-conjugated monoclonal antibodies: CD4-APC-H7, CXCR3-PE, CCR6-BB515, PD-1-PE-Cy7 and ICOS-BV450 (all from BD biosciences, San José, CA, USA)  (Mallett et al., 2019; Mousset et al., 2019). Acquisition of PBLs was performed with a FACS CANTO II flow cytometry (BDbiosciences) and analyzed with Flow-Jo software v10.6.2. Gating strategy is shown (**Figure S1**).

Cells CXCR3+/CCR6- gated from CD4 were considered Th1 cells, CXCR3-/CCR6+ cells were considered as Th17 and CXCR3-/CCR6- as Th2. Regarding the activation grade, cells PD-1-/ICOS- were considered as quiescent Th cells, PD-1-/ICOS+ cells as early-activated cells, PD-1+/ICOS+ as late activation markers and PD-1+/ICOS- as exhausted or senescent cells (Mallett et al., 2019).

### Cytokine Profile Assay

Serum cytokine Th profile was determined by Human Cytokine Magnetic Bead Panel Kit (EMD Millipore Corporation) using a LABScan™ 100 Luminex. The results were analyzed with Luminex xPONENT42 softwarev3.1. Cytokines included were IL-2, IL-4, IL-6, IL-10, IL-12p70, IL-13, IL-15, INF-gamma, IP-10, MCP-1, and VEGFα.

### Statistical Analysis

Results of qualitative variables were expressed as absolute frequency and percentage. Results of the scaled variables were expressed as median with interquartile range. Comparison between qualitative variables was evaluated with Pearson’s Chi-square test or Fisher’s exact test, when appropriate.

Comparison between scaled variables with two categories were performed using Mann-Whitney’s U test, whereas variables with more than two categories were analyzed using Kruskal-Wallis test.

The odds ratio was used to express the relative measure of an effect between a binary outcome variable and a predictor variable. Multivariate analysis was performed using logistic regression model. Significant results were considered with a p-value < 0.05.

Data were analyzed with MedCalc ^®^ version 19.3 (MedCalc Software, Ostend, Belgium).

## Results

### Patient’s Characteristics

Median age of the COVID-19 cohort was 55 years (IQR: 44–75.25) with a higher proportion of males (67%). No significant age differences were found when COVID-19 patients were compared with the reference population (55 vs. 50, respectively, p = 0.195). Sex distribution was also analyzed between groups, no significant differences being found (**Table 1**).

**Table 1:** Age and sex comparison between reference population and COVID-19 cohort.

Variables	Reference population N = 21	COVID-19 patients N = 55	p-value*	COVID-19 patients groups N = 55			
				Deceased; N = 9	Alive; N = 46	p-value**	p-value***
**Age**	50 (43.75–59.25)	55 (44–75.25)	0.195	87 (82.25–91.75)	53 (44–62)	<0.001	<0.001
**Sex**	–	–	–	–	–	–	–
** Female**	8 (38%)	18 (33%)	0.738	2 (22%)	16 (35%)	0.514	0.748
** Male**	13 (62%)	37(67%)		7 (78%)	30 (65%)		

*Reference population vs. COVID-19 cohort; **Reference population vs. Deceased COVID-19 patients; ***Alive COVID-19 patients vs. Death COVID-19 patients.

COVID-19 patients were classified according to their most critical event during the evolution of the disease (**Figure 1** **)**.

Deceased patients in COVID-19 group were significantly older than the reference population (p < 0.001) and COVID-19 survival patients (p < 0.001). No significant differences were observed in sex distribution (**Table 1**). Similarly, when laboratory parameters were compared among COVID-19 groups, the total number of lymphocytes were significantly depleted in deceased patients compared to survival patients (p = 0.014). The patients who died also presented a high rate of lymphopenia (p = 0.013) and higher D-dimer levels (p = 0.011). No significant differences were found regarding LDH concentration, CRP levels, ferritin, % RT-PCR or serology, and the development of ARDS (**Table 2**).

**Table 2:** Laboratory parameters compared among COVID-19 groups show median and IQR.

Variables	COVID-19 patients groups N = 55				p-value
	G1-Deceased; N = 9	G2-ICU; N = 7	G3-Immunomodulators; N = 14	G4-Benign Course; N = 25	
**Biochemical Markers**	–	–	–	–	–
** D-Dimer**	4196 (1105–14116)	1714 (1162–1886)	628 (554.25–822)	579.5 (298–959)	0.011
** LDH**	206.5 (287–338)	314.5 (271.5–370.5)	345 (255–411)	279 (226.25–362)	0.369
** CRP**	8.29(5.51–24.24)	4 (1.07–20.37)	6.05 (3.94–13.92)	4.49 (1.3–11.1)	0.169
** Ferritin**	963.4 (535.4–1569.5)	1268 (700.1–1837)	1014 (533.6–1574.9)	646.9 (283.75–1146.35)	0.319
**Immunological Markers**	–	–	–	–	–
** Lymphocyte**	650 (500–1,050)	1500 (800–2,600)	1550 (1400–2,000)	1300 (775–1,855)	0.014
** Lymphopenia**	5 (55.5%)	3 (43%)	0 (0%)	8 (32%)	0.013
**Diagnosis**	–	–	–	–	–
** RT-PCR**	8 (89%)	6 (86%)	9 (64%)	17 (68%)	0.463
** Serology**	1 (11%)	1 (14%)	5 (36%)	8 (32%)	0.392
**ARDS**	4 (44.5%)	4 (57%)	5 (36%)	7 (28%)	0.351

LDH, lactate dehydrogenase; CRP, C-reactive protein; RT-PCR, retrotranscriptase polymerase chain reaction; ARDS, acute respiratory distress syndrome; ICU, intensive care unit.

### Differences in Th Subsets Between Reference Population and COVID-19 Patients

When analyzing the differences in the Th subsets between in COVID-19 patients and controls, it was found that the percentage of CD4+ T lymphocytes with Th1 and Th17 phenotype was significantly reduced in the cells of COVID-19 patients (2.99% vs. 6.68%, p < 0.001 for Th1; and 3.41% vs. 6.95%, p = 0.012 for Th17).

Quiescent Th cells were analyzed when between COVID-19 patients and healthy controls. The percentages of quiescent Th1 cells (6% vs. 9.76% in controls, p = 0.007). Percentage of Th17 and Th2 tends to be reduced in COIVD-19 patients (10.1% vs. 13.2%, p = 0.19 for Th17; 21.3% vs. 35.9%, p = 0.111 for Th2).

The percentage of early-activated cells in the Th1 and Th17 subsets were reduced compared with healthy controls (0.65% vs. 1.17%, p = 0.026 for Th1; 0.8% vs. 1.19%, p = 0.091 for Th17). The percentage of Th17 was significantly depleted in COVID-19 patients versus controls (0.58% vs. 1.44%, p = 0.007) regarding the late activity phenotype. The activated status was augmented in Th2 subset (17.6% in COVID-19 patients vs. 1.31% in healthy controls, p = 0.004).

COVID-19 patients presented a reduced percentage of senescent Th2 cells compared to controls (4.7% vs. 11.5%, p < 0.001)

No significant differences were shown in **Table S1**.

### Th Cell Subsets Imbalance According to the Disease Progression

After examining the Th cell subsets differences between COVID-19 patients and healthy controls, the COVID-19 groups were compared according to disease evolution. No significant differences in the percentage of Th1, Th2 or Th17 and activation grade were observed in the COVID-19 patient groups.

Deceased COVID-19 patients showed a lower percentage of quiescent Th1 cells compared to COVID-19 patients with a benign course of the disease (p = 0.016). Increased percentage of senescent Th1 cells tends to be associated with a benign course of the disease. However, higher proportions of senescent Th2 were associated with death (p = 0.009).

**Figure 2:**
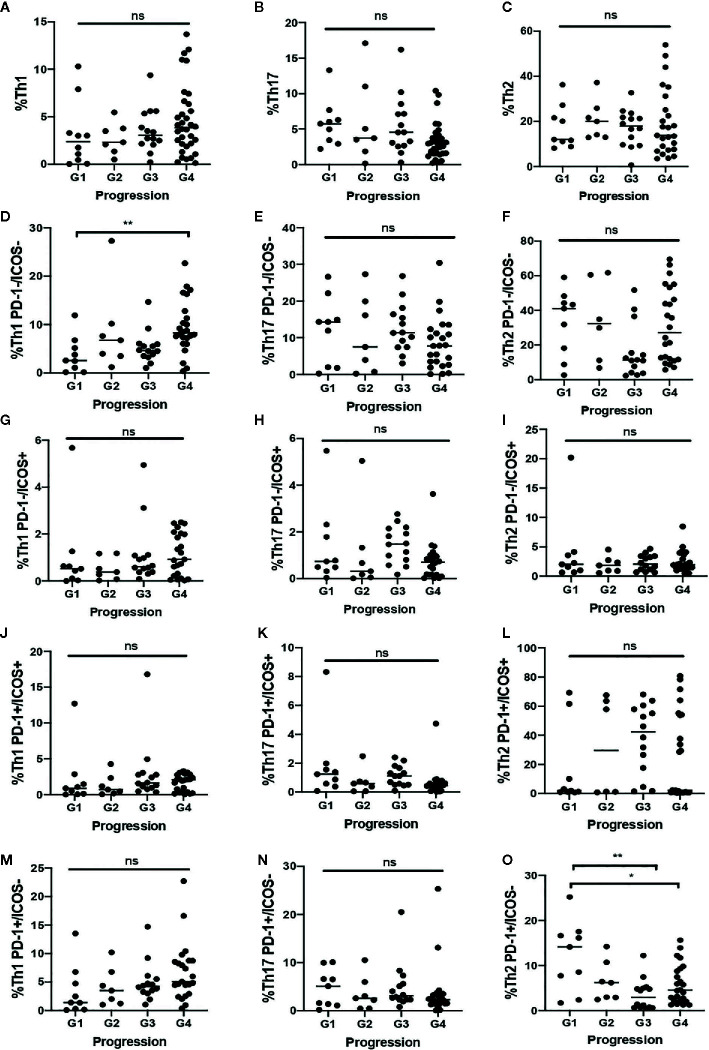
Th distribution in COVID-19 patients according to the most critical event during disease. **A–C)** Total proportion of Th 1 **(A)**. Th17 **(B)** and Th2 **(C)**. **(D, F)** Proportion of quiescent Th1 **(D)**, Th17 **(E)** and Th2 **(F)**. **(G–I)** Proportion of early activated Th1 **(G)**. Th17 **(H)** and Th2 **(I)**. **(J–L)** Proportion of late activated Th1 **(J)**, Th17 **(K)**, and Th2 **(L)**. **(M–O)** Proportion of senescent Th1 **(M)**, Th17 **(N)**, and Th2 **(O)**. G1: Death; G2: Intensive Care Unit; G3: Immunomodulators; G4: Benign-Course. *. <0.05 **. <0.01 ns, not significant.

There were no significant results regarding total proportion of Th, the grade activation and the senescent status (**Figure 2** **)**.

### Differences in Th Subsets Between COVID-Like and COVID-19 Patients

Comparing the Th subsets between COVID-Like and COVID-19 patients, no significant differences were found in the total proportion of Th1, Th17, and Th2.

COVID-19 patients presented a significantly higher percentage of late-activated Th2 cells than COVID-19 patients (17.6% vs. 0.76%, p = 0.006). The result of this comparation is shown in **Table S2**. As can be seen in **Figure 2**, when the COVID-Like patients were compared with the four subgroups of COVID-19 patients, their similarity with the COVID patients with good progression (groups 3 and 4) stands out.

### Death-Associated Factors

Th subsets were evaluated in relationship with mortality (**Table 3**). The two factors that conferred risk in the univariate analysis, that is, the total number of lymphocytes (p = 0.018) and the senescent Th2 percent (p = 0.021), were included in a multivariate analysis. The total number of lymphocytes (OR 0.15, 95% CI: 0.02–0.81, p = 0.027) and the higher senescent percentage of Th2 cells (OR: 13.88, 95% CI: 1.33–143.88, p = 0.027) behaved like independent and significant risk factors for death with an area under the ROC curve of 0.879 (95% CI: 0.760–0.952) (**Table 3**).

**Table 3:** Univariate and multivariate analyses associated to death risk with all significant variables.

VARIABLES	p-value	OR	OR 95% CI	AUC	AUC 95% CI	p-value	OR	OR 95% CI
**Lymphocytes**	0.018	0.2	0.05–0.076	0.77	0.634–0.873	0.027	0.15	0.02–0.81
**% Th1**	0.514	0.65	0.18–2.33	0.55	0.413–0.687	–	–	–
**%Th17**	0.085	3.8	0.83–17.37	0.63	0.495–0.761	–	–	–
**% Th2**	0.777	0.86	0.31–2.38	0.52	0.385–0.661	–	–	–
**%Th1 PD-1-/ICOS**−	0.067	0.28	0.07–10.9	0.68	0.545–0.803	–	–	–
**%Th17 PD-1-/ICOS**−	0.257	2.38	0.53–10.69	0.6	0.464–0.734	–	–	–
**%Th2 PD-1-/ICOS**−	0.259	27.778	0.47–16.35	0.618	0.451–0.766	–	–	–
**% Th1 PD-1-/ICOS+**	0.710	0.72	0.13–3.96	0.53	0.391–0.666	–	–	–
**%Th17 PD-1-/ICOS+**	0.574	1.53	0.34–6.31	0.55	0.411–0.685	–	–	–
**%Th2 PD-1-/ICOS+**	0.161	2.57	0.68–9.69	0.58	0.429–0.712	–	–	–
**%Th1 PD-1+/ICOS+**	0.631	1.79	0.14–19.46	0.52	0.384–0.659	–	–	–
**%Th17 PD-1+/ICOS+**	0.238	5.6	0.31–99.41	0.54	0.405–0.68	–	–	–
**%Th2 PD-1+/ICOS +**	0.09	0.24	0.04–1.28	0.66	0.52–0.783	–	–	–
**%Th1 PD-1+/ICOS**−	0.750	0.69	0.07–6.47	0.52	0.382–0.657	–	–	–
**%Th17 PD-1+/ICOS**−	0.073	3.86	0.87–16.97	0.656	0.514–0.780	–	–	–
**%Th2 PD-1+/ICOS**−	0.021	7.23	1.33–39.12	0.72	0.589–0.837	0.027	13.88	1.33–143.88
**Area Under the Curve**						0.879	0.760–952	

PD-1-/ICOS−, Quiescent; PD-1-/ICOS+, early-activated; PD-1+/ICOS+, late-activated; PD-1+/ICOS, senescent.

### Cytokine Profiling

The cytokine serum profile was compared between COVID-19 patients and healthy controls. An increased expression of IL-2 (p < 0.001) and INF-gamma (p = 0.024) and a reduction of IL-12p70 (p < 0.001) in COVID-19 patients were observed regarding the Th1 cytokines. Regarding Th2 cytokines, COVID-19 patients showed higher levels of IL-6, IL-10 and IL-13, but lower levels of IL-4 (p < 0.001, for all of them). Other cytokines like IL-15 and IP-10 presented significantly increased levels in COVID-19 compared to healthy donors (p < 0.001, in both cases). No significant differences were observed in relationship to MCP-1 and VEGFα (**Table S3**).

Cytokine levels were studied according to the worst event during hospitalization, without any significant results among groups (**Figure S3**).

Patients who died presented significantly higher levels of IL-15: 9.65 pg/ml, IQR: 7.5–15.3 vs. 5.06 pg/ml, IQR: 2–8.18; (p = 0.036). A marked trend was observed when MCP-1, IL-6 and IL-10 were analyzed to describe which ones of the higher levels were associated to poorer evolution of the disease, but these differences did not achieve statistical significance (**Table 4**).

**Table 4:** Cytokine profile differences in alive and deceased patients.

Cytokines	Alive; N = 28		Death; N = 6		p-value
	Median	IQR	Median	IQR	
**IL-2**	0.1	0.1–0.85	0.29	0.1–0.86	0.573
**IL-4**	1.4	1.4–5.56	1.4	1.4–381.12	0.827
**IL-6**	14.33	2.12–41.49	39.55	8.6–69.36	0.258
**IL-10**	8.78	3.2–18.28	17.19	00.18–26.84	0.113
**IL-12p70**	0.05	0.05–0.44	0.05	0.05–0.08	0.667
**IL-13**	0.5	0.5–1.61	0.55	0.5–16.04	0.46
**IL-15**	5.05	2–8.18	9.65	7.5–15.3	0.036
**INFg**	0.39	0.3–8.77	5.15	2.24–7.18	0.314
**IP-10**	1183.65	296.29–3151.72	1358.33	876.82–3858.76	0.588
**MCP-1**	527.91	358.96–889.92	1248.8	535.58–1999.45	0.064
**VEGFα**	92.73	17.45–297.8	75.84	16–93.99	0.401

## Discussion

We have demonstrated in this study that a Th1 coordinated immune response during the infection by SARS-CoV-2 associates with good prognosis and resolution of COVID-19. The type of Th response impacted the disease outcome, since 78% of patients who finally died had produced an overreactive Th2 response. The type of Th response has been demonstrated to be crucial for the resolution of the disease (Infante-Duarte, 1999; Spellberg and Edwards, 2001).

We have not been able to observe changes in activated cells because they are localized in tissues controlling the infection. On the other hand, those that have already ended their task abandon the tissue and migrate into the blood (Morris, 1998). The detection of senescent Th cells in blood could be an indicator of the type of the past immune response.

In intracellular *M. tuberculosis* infection, Bacillus Calmette-Guerin (BCG) vaccine promotes a Th1 response, protecting human beings from tuberculosis development. The utility of BCG vaccine has been studied in COVID-19 patients with very surprising results. Lower mortality and infective rates were observed in vaccinated patients, who produced Th1 responses (Ozdemir et al., 2020). Our study has highlighted the importance of Th1 hypoactivation and Th2 overreaction, with subsequent exhaustion, associated with poorer prognosis. A possible hypothesis to explain the severe forms of COVID-19 is that the Th2 responses prevail in those patients, producing an ineffective response to intracellular pathogens like viruses. However, contrary to other studies, which propose that a higher % of Th2 cells is associated with critical care and poor prognosis (Wei et al., 2020), we did not observe any differences when we analyzed the total % of Th1, Th2, and Th17 between the different clinical course of the disease. This finding could suggest that the activation grade could be a more important prognostic factor than the total percentage of each Th as we have observed that patients with a more benign course of the disease tend to present higher % of senescent Th1 cells as a consequence of their rapid activation. The absence of significance could be due to the small size of each group.

When we compared the patients with COVID-19 with the healthy controls, we found that the majority of the patients not only presented a general reduction in the total percentage of Th but also in their degree of activation. This clear reduction could be related to the common lymphopenia (Wang et al., 2020; Yang et al., 2020). We have observed that COVID-19 patients had an overactive Th2 response against the virus. Therefore, compared to COVID-like patients, COVID-19 patients also had an overactive Th2 response.

Observations from the COVID-Like group should be handled with caution. The World Health Organization has reported that in pandemic situations it is not possible to rule out that patients who have had symptoms similar to COVID and RT-PCR negative had the disease (WHO, 2020). It is possible that some patients in the COVID-Like group were seronegative COVID-19 patients with a mild form of the disease. In this way, it is worth highlighting the similarities observed between the patients in this group and the COVID-19 patients with good evolution.

The mortality rate described in COVID-19 is highly variable: from 2.2% to 15% in some studies (Cao et al., 2020; Chen et al., 2020; Huang C. et al., 2020; Rothan and Byrareddy, 2020; Wang et al., 2020). The factors associated with high mortality in COVID-19 are only partially known (obesity, age, previous illnesses, etc.) (Wolff et al., 2020; Zheng et al., 2020).

Our results have shown that total number of lymphocytes and higher exhausted Th2 are independent risk factors for death. Our findings are consistent with that previously described in SARS-Cov (Li et al., 2008; Vabret et al., 2020). Lymphopenia was an important finding as similar results were observed in SARS-Cov and MERS-Cov (Peiris et al., 2003; Wong et al., 2003; Erickson et al., 2013; Ko et al., 2016; Min et al., 2016). The presence of higher exhausted Th2 suggests an over-activation with the consequent exhaustion, a phenomenon that has been observed in CD8+ cell. All this activation is driven by rapid viral replication and its exposure to lymphocytes (Ko et al., 2016; Min et al., 2016). Th2 hyperactivation could explain why severe patients have higher antibody titers than mild or asymptomatic patients, as their cytokines stimulate antibody production (Spellberg and Edwards, 2001).

We have observed that the SARS-Cov2 infection is characterized by a pro-inflammatory environment with increased levels of IL-2, IL-6, IL-15, IP-10, and INF-gamma. As described previously, cytokines are important for the clearance of respiratory infections (Kimura et al., 2013).

Our results have shown that patients with a poor prognosis present a Th cytokine profile associated with changes in the inflammatory status, as previously described (Lucas et al., 2020), with a slight increase in the expression of IL-6, IL-10, MCP-1, and INF-gamma and a marked increase of IL-15.

High levels of these cytokines have been reported in publications to be related with a worse respiratory status (Vabret et al., 2020; Yao et al., 2020). It has also been described that critical respiratory manifestations have correlated with high levels of IL-6 and IL-15, this being an important predictor of disease progression (Leahy et al., 2016; Rocio et al., 2020). High levels of IL-6, MCP-1 and IL-15 correlated to poor prognosis in SARS-Cov and MERS-Cov infections, acting as hypercytokinemia and systemic syndrome predictors, have also been reported (Wang et al., 2004; Wong et al., 2004; Zhang et al., 2004; Li et al., 2020). Another factor which could help to understand Th imbalance in COVID-19 is IL-10, a potent inhibitor of IL-12 and Th1 response. The significance of the presence of IL10 in COVID-19 patients has been extensively described in the literature, this suggesting that IL-10 could discriminate disease progression (Gong et al., 2020; Zhou et al., 2020).

IL-15 is a cytokine secreted by different cell types like endothelial cells, respiratory epithelium or neurons. It matures in macrophages and dendritic cells and plays a pleiotropic role in innate and adaptive immunity. Similarly to IL-2, IL-15 is a very important molecule in NK and T lymphocyte activation and proliferation like IL-2 (Perera et al., 2012). Activated T cell recruitment and contraction in inflammatory tissues, enriched with antigenic molecules, is a crucial event in the immune response, which principally depends on antigenic presentation by endothelial cells from swollen vessels (Ward et al., 2017). IL-15 has been identified as an important mediator in T cell and NK migration to infected tissue, due to IL-15 improves LFA-1/ICAM-1 binding affinity in endothelial cells (Oppenheimer-marks et al., 1998). We hypothesize that its elevation is due to an additional effort of the immune system in T lymphocyte activation in an alternate way, as a compensatory mechanism to substitute an inefficient Th1 response.

Nowadays, there is extensive state of the art knowledge regarding the causes of lymphopenia. Some authors have stated that lymphopenia is caused by direct infection of lymphocyte, as they express ACE2 in their surface (Xu et al., 2020). Another hypothesis states that the immune system hyperactivation could lead to lymphocyte apoptosis by inflammatory cytokines (Liao et al., 2002; Sähr et al., 2015). On the other hand, other publications support the idea that immune system dysregulation in COVID-19 leads to a “cytokine storm,” suggesting a polyclonal activation of T lymphocytes, possibly associated to superantigens (Cossarizza, 1997). Others have reported that the lymphopenia associated with COVID-19 could be a consequence of pulmonary recruitment and entrapment of lymphocytes, due to modification of adhesion molecules (CD44, LFA-1 and ICAM-1) (Morris, 1998; Bohn et al., 2020). Recently, Roncati et al. published that SARS-Cov2 is able to infect lymphocytes *via* dipeptidyl peptidase-4 (DPP-4) receptor, which is overexpressed in Th1 lymphocyte rather in Th2 (Willheim et al., 1997; Annunziato et al., 1998; Sakata-Kaneko et al., 2000). This finding could support our results as we have observed that COVID-19 patients have reduced total %Th1 compared with healthy controls. Moreover, the total %Th2 was slightly higher in COVID-19 patients.

When a Th effector response is polarized to Th2, antibody production is not only stimulated but the cell-mediated immunity is also suppressed (Kaiko et al., 2007). This fact is very important regarding the treatment that patients receive. In many cases, glucocorticoids are chosen as a treatment of choice in this novel condition, but they are a double-edged sword as they act as immunosuppressant reducing IL-2, which is essential for Th1 responses, where only Th2 are able to proliferate (Krakauer, 1995; Larsson and Linden, 1998; Ramírez et al., 1998; Vieira et al., 1998; Franchimont et al., 2000), this severely affecting the patients.

Recently, some authors have proposed that patients susceptible to severe lung pathology in COVID-19 may have the highest Th17. This response could be promoted by IL-6 and may be accompanied by eosinophilia. In our patients, Th17 was significantly reduced compared to controls, but no differences were found according to clinical presentations of the disease (Hotez et al., 2020; Wu and Yang, 2020).

Several limitations exist in this study. First of all, it is limited by the reduced sample size of our study so that these markers not only need to be evaluated in a larger cohort but also further independent studies are needed to validate our results. Secondly, in order to perform a multivariate analysis, due to the few events that were of interest in this study, the univariate and multivariate evaluation could not be carried out with raw data, thus requiring each variable to be transformed into ranges by histogram of frequency analysis (**Table S4**). Another limitation is that we studied the cytokine profile of each patient in sera but not in cell culture and we have not studied antibody titration to examine its association to prognosis. Finally, using blood donors is a disadvantage regarding age as severe forms of the disease usually affect patients older than 65 in whom blood donation is not recommended.

In summary, although Th1/Th2 imbalance related to poor prognosis is well known in infections of different etiology, much remains to be known in COVID-19 patients. At present, most of the effects are directed toward reducing an exacerbated inflammatory response. Therefore, the search for new therapies, which redirect Th2 responses to Th1, may tip the scale toward less severe forms of the disease.

## Data Availability Statement

The raw data supporting the conclusions of this article will be made available by the authors, without undue reservation.

## Ethics Statement

The studies involving human participants were reviewed and approved by Clinical Research Ethics Committee of University Hospital 12 de Octubre (reference no. 20/167). The patients/participants provided their written informed consent to participate in this study.

## Author Contributions

AS and EP-A developed the theory. AS and FG-E designed the study and planned the experiments. PS-F, LN, DA, SG and FG-E carried out the experiments. AL, DP, and RD-S supported the clinical aspects of the study. FG-E analyzed the data and took the lead in writing the manuscript. AS, EP-A, EM, LA and OC-M reviewed the final manuscript. All authors provided critical feedback and helped shape the research, analysis and manuscript. All authors contributed to the article and approved the submitted version.

## Funding

This work was supported by grants: COVID-19 Research Call COV20/00181 and PI17-0147 from Institute of Health Carlos III from Spanish Ministry of Science and Innovation, co‐financed by European Development Regional Fund “A way to achieve Europe”.

## Conflict of Interest

The authors declare that the research was conducted in the absence of any commercial or financial relationships that could be construed as a potential conflict of interest.

## References

[B1] Alberts B. Johnson A. Lewis J. Raff M. Roberts K. Walter P. (2002). Molecular Biology of the Cell. Helper T Cells and Lymphocyte Activation. 4th edition, New York: Garland Science. Available at: https://www.ncbi.nlm.nih.gov/books/NBK26827/.

[B2] Annunziato F. Galli G. Cosmi L. Romagnani P. Manetti R. Maggi E.. (1998). Molecules Associated with Human Th1 or Th2 Cells. Eur. Cytokine Netw. 9 (3 Suppl), 12–16. 9831180

[B3] Bohn M. K. Hall A. Sepiashvili L. Jung B. Steele S. Adeli K. (2020). Pathophysiology of COVID-19 : Mechanisms Underlying Disease Severity and Progression. Physiology (Bethesda) 89, 288–301. 10.1152/physiol.00019.2020 PMC7426542 32783610

[B4] Buszko M. Park J. H. Verthelyi D. Sen R. Young H. A. Rosenberg A. S. (2020). The Dynamic Changes in Cytokine Responses in COVID-19: A Snapshot of the Current State of Knowledge. Nat. Immunol. 21 (10), 1146–1151. 10.1038/s41590-020-0779-1 32855555

[B5] Calarota S. A. Weiner D. B. (2004). Enhancement of Human Immunodeficiency Virus Type 1-DNA Vaccine Potency through Incorporation of T-Helper 1 Molecular Adjuvants. Immunol. Rev. I (99), 84–99. 10.1111/j.0105-2896.2004.00150.x 15233728

[B6] Cao B. Wang Y. Wen D. Liu W. Wang J. Fan G.. (2020). A Trial of Lopinavir–Ritonavir in Adults Hospitalized with Severe Covid-19. New Engl. J. Med. 382 (19), 1787–1799. 10.1056/NEJMoa2001282 32187464 PMC7121492

[B7] Chen N. Zhou M. Dong X. Qu J. Gong F. Han Y.. (2020). Epidemiological and Clinical Characteristics of 99 Cases of 2019 Novel Coronavirus Pneumonia in Wuhan, China: A Descriptive Study. Lancet 395 (10223), 507–513. 10.1016/S0140-6736(20)30211-7 32007143 PMC7135076

[B8] Chen Z. Wherry E.J. (2020). T Cell Responses in Patients with COVID-19. Nat. Rev. Immunol. 20 (9), 529–536. 10.1038/s41577-020-0402-6 32728222 PMC7389156

[B9] Clerici M. Shearer G. M. (1993). A TH1→TH2 Switch Is a Critical Step in the Etiology of HIV Infection. Immunol. Today Immunol. Today 14 (3), 107–111. 10.1016/0167-5699(93)90208-3 8096699

[B10] Cossarizza A. (1997). T-Cell Repertoire and HIV Infection. AIDS 11, 1075–1088. 10.1097/00002030-199709000-00001 9233453

[B11] Diao Bo. Wang C. Tan Y. Chen X. Liu Y. Ning L.. (2020). Reduction and Functional Exhaustion of T Cells in Patients With Coronavirus Disease 2019 (COVID-19). Front. Immunol. 11, 827. 10.3389/fimmu.2020.00827 32425950 PMC7205903

[B12] Erickson J. J. Gilchuk P. Hastings A. K. Tollefson S. J. Johnson M. Downing M. B.. (2012). Viral Acute Lower Respiratory Infections Impair CD8 + T Cells through PD-1. J. Clin. Invest. 122 (8), 2967–2982. 10.1172/JCI62860 22797302 PMC3408742

[B13] Franchimont D. Galon J. Gadina M. Visconti R. Zhou Y.-J. Aringer M.. (2000). Inhibition of Th1 Immune Response by Glucocorticoids: Dexamethasone Selectively Inhibits IL-12-Induced Stat4 Phosphorylation in T Lymphocytes. J. Immunol. 164 (4), 1768–1774. 10.4049/jimmunol.164.4.1768 10657623

[B14] Gao Z. Xu Y. Sun C. Wang X. Guo Y. Qiu S.. (2020). A Systematic Review of Asymptomatic Infections with COVID-19. J. Microbiol. Immunol. Infect. 10.1016/j.jmii.2020.05.001 PMC7227597 32425996

[B15] Gong J. Dong H. Xia S. Q. Huang Y. Z. Wang D. Zhao Y.. (2020). Correlation Analysis Between Disease Severity and Inflammation-Related Parameters in Patients with COVID-19 Pneumonia. BMC Infect. Dis 20 (963), 2020.02.25.20025643. 10.1101/2020.02.25.20025643 PMC7750784 33349241

[B16] Guan W. J. Ni Z. Y. Hu Y. Liang W. H. Ou C. Q. He J. X.. (2020). Clinical Characteristics of Coronavirus Disease 2019 in China. New Engl. J. Med. 382 (18), 1708–1720. 10.1056/NEJMoa2002032. April. 32109013 PMC7092819

[B17] Guo Y. R. Cao Q. D. Hong Z. S. Tan Y. Y. Chen S. D. Jin H. J.. (2020). The Origin, Transmission and Clinical Therapies on Coronavirus Disease 2019 (COVID-19) Outbreak- A n Update on the Status. Mil. Med. Res. 7 (1), 11. 10.1186/s40779-020-00240-0 32169119 PMC7068984

[B18] Hotez P. J. Bottazzi M. E. Corry D. B. (2020). The Potential Role of Th17 Immune Responses in Coronavirus Immunopathology and Vaccine-Induced Immune Enhancement. Microbes Infect. 22 (4–5), 165–167. 10.1016/j.micinf.2020.04.005 32305501 PMC7162764

[B19] Huang W. Berube J. Mcnamara M. Saksena S. Hartman M. Arshad T.. (2020) Lymphocyte Subset Counts in COVID-19 Patients: A Meta-Analysis. Cytometry Part A. Cytometry Part A 97 (8), 772–776. 10.1002/cytoa.24172 PMC7323417 32542842

[B20] Huang C. Wang Y. Li X. Ren L. Zhao J. Hu Y.. (2020). Clinical Features of Patients Infected with 2019 Novel Coronavirus in Wuhan, China. Lancet 395 (10223), 497–506. 10.1016/S0140-6736(20)30183-5 31986264 PMC7159299

[B21] Hurwitz J. L. (2020). B Cells, Viruses, and the SARS-COV-2/COVID-19 Pandemic of 2020. Viral Immunol. 33 (4), 251–252. 10.1089/vim.2020.0055 32348715 PMC7247029

[B22] Infante-Duarte C. (1999). Th1/Th2 Balance in Infection., in Springer Seminars in Immunopathology. Germany: Springer Verlag. 10.1007/BF00812260 10666776

[B23] Kaiko G. E. Horvat J. C. Beagley K. W. (2007). Immunological Decision-Making : How Does the Immune System Decide to Mount a Helper T-Cell Response? Immunology 123 (3), 326–338. 10.1111/j.1365-2567.2007.02719.x 17983439 PMC2433332

[B24] Kamperschroer C. Quinn D. G. Kamperschroer C. Quinn D. G. (2020). The Role of Proinflammatory Cytokines in Wasting Disease During Lymphocytic Choriomeningitis Virus Infection. J. Immunol. 169 (1), 340–349. 10.4049/jimmunol.169.1.340 12077263

[B25] Kim G. U. Kim M. J. Ra S. H. Lee J. Bae S. Jung J.. (2020). Clinical Characteristics of Asymptomatic and Symptomatic Patients with Mild COVID-19. Clin. Microbiol. Infect. 26 (7), 948.e1–948.e3. 10.1016/j.cmi.2020.04.040 32360780 PMC7252018

[B26] Kimura H. Yoshizumi M. Ishii H. Oishi K. Ryo A. (2013). Cytokine Production and Signaling Pathways in Respiratory Virus Infection. Front. Microbiol. 4, 2013.00276 (SEP). 10.3389/fmicb.2013.00276 PMC3774987 24062733

[B27] Ko J. H. Park G. E. Lee J. Y. Lee J. Y. Cho S. Y. Ha Y. E.. (2016). Predictive Factors for Pneumonia Development and Progression to Respiratory Failure in MERS-CoV Infected Patients. J. Infect. 73 (5), 468–475. 10.1016/j.jinf.2016.08.005 27519621 PMC7112644

[B28] Krakauer T. (1995). Differential Inhibitory Effects of Interleukin-10, Interleukin-4 and Dexamethasone on Staphylococcal Enterotoxin-Induced Cytokine Production and T Cell Activation. J. Leuk. Biol. 57 (3), 450–454. 10.1002/jlb.57.3.450 7884317

[B29] Larsson S. Linden M. (1998). Effects of a Corticosteroid, Budesonide, on Production of Bioactive IL-12 by Human Monocytes. Cytokine 10 (10), 786–789. 10.1006/cyto.1998.0362 9811532

[B30] Leahy T.R. McManus R. Doherty D. G. Grealy R. Coulter T. Smyth P.. (2016). Interleukin-15 Is Associated with Disease Severity in Viral Bronchiolitis. Eur. Respir. J. 47 (1), 212–222. 10.1183/13993003.00642-2015 26541527

[B31] Li C. K.-f. Wu H. Yan H. Ma S. Wang L. Zhang M.. (2008). T Cell Responses to Whole SARS Coronavirus in Humans. J. Immunol. 181 (8), 5490–5500. 10.4049/jimmunol.181.8.5490 18832706 PMC2683413

[B32] Li S. Jiang L. Li X. Lin F. Wang Y. Li B.. (2020). Clinical and Pathological Investigation of Patients with Severe COVID-19. JCI Insight 5 (12), 1–13. 10.1172/jci.insight.138070 PMC7406259 32427582

[B33] Liao Y.-C. Liang W.-G. Chen F.-W. Hsu J.-H. Yang J.-J. Chang M.-S. (2002). IL-19 Induces Production of IL-6 and TNF-α and Results in Cell Apoptosis Through TNF-α. J. Immunol. 169 (8), 4288–4297. 10.4049/jimmunol.169.8.4288 12370360

[B34] Lucas C. Wong P. Klein J. Tiago B. R. C. Silva J. Sundaram M.. (2020). Longitudinal Analyses Reveal Immunological Misfiring in Severe COVID-19. Nature 584 (7821), 463. 10.1038/s41586-020-2588-y 32717743 PMC7477538

[B35] Mallett G. Laurence A. Amarnath S. (2019). Programmed Cell Death-1 Receptor (Pd-1)-Mediated Regulation of Innate Lymphoid Cells. Int. J. Mol. Sci. 20 (11), 1–13. 10.3390/ijms20112836 PMC6601034 31212601

[B36] Mescher M. F. Curtsinger J. M. Agarwal P. Casey K. A. Gerner M. Hammerbeck C. D.. (2006). Signals Required for Programming Effector and Memory Development by CD8+ T Cells. Immunol. Rev. 211 (June), 81–92. 10.1111/j.0105-2896.2006.00382.x 16824119

[B37] Min C. K. Cheon S. Ha N. Y. Sohn K. M. Kim Y. Aigerim A.. (2016). Comparative and Kinetic Analysis of Viral Shedding and Immunological Responses in MERS Patients Representing a Broad Spectrum of Disease Severity. Sci. Rep. 6 (1), 1–12. 10.1038/srep25359 27146253 PMC4857172

[B38] Morris O. (1998). Expression of T Lymphocyte Adhesion Molecules: Regulation during Antigen-Induced T Cell Activation and Differenciation. Crit. Reves Immunol. Immunol. 18, 153–184. 10.1615/critrevimmunol.v18.i3.10 9637409

[B39] Mosmann T. R. Sad S. (1996). The Expanding Universe of T-Cell Subsets: Th1, Th2 and More. Immunol. Today 17 (3), 138–146. 10.1016/0167-5699(96)80606-2 8820272

[B40] Mousset C. M. Hobo W. Woestenenk R. Preijers F. Dolstra H. van der Waart A. B. (2019). Comprehensive Phenotyping of T Cells Using Flow Cytometry. Cytometry Part A 95 (6), 647–654. 10.1002/cyto.a.23724 30714682

[B41] Neidleman J. Luo X. Frouard J. Xie G. Gill G. Stein E. S.. (2020). SARS-CoV-2-Specific T Cells Exhibit Phenotypic Features of Robust Helper Function, Lack of Terminal Differentiation, and High Proliferative Potential. Cell Rep. Med. 1 (6):100081. 10.1016/j.xcrm.2020.100081 32839763 PMC7437502

[B42] Oppenheimer-marks N. Brezinschek R. I. Mohamadzadeh M. Randi V. Peter E.L. (1998). Interleukin 15 Is Produced by Endothelial Cells and Increases the Transendothelial Migration of T Cells In Vitro and in the SCID Mouse – Human Rheumatoid Arthritis Model In Vivo. J. Clin. 101 (6), 1261–1272. 10.1172/JCI1986 PMC508680 9502767

[B43] Ozdemir C. Kucuksezer U. C. Tamay Z. U. (2020). Is BCG Vaccination Affecting the Spread and Severity of COVID-19?” Allergy 75 (7), 1824–1827. 10.1111/all.14344 32330314

[B44] Peiris J. S. M. Lai S. T. Poon L. L. M. Guan Y. Yam L. Y. C. Lim W.. (2003). Coronavirus as a Possible Cause of Severe Acute Respiratory Syndrome. Lancet 361 (9366), 1319–1325. 10.1016/S0140-6736(03)13077-2 12711465 PMC7112372

[B45] Perera P.-y. Lichy J. H. Waldmann T. A. Perera L. P. (2012). The Role of Interleukin-15 in Inflammation and Immune Responses to Infection : Implications for Its Therapeutic Use. Microbes Infect. 14 (3), 247–261. 10.1016/j.micinf.2011.10.006 22064066 PMC3270128

[B46] Qin C. Zhou L. Hu Z. Zhang S. Yang S. Tao Y.. (2020). Dysregulation of Immune Response in Patients with COVID-19 in Wuhan, China. Clin. Infect. Dis. 71 (15), 762–768. 10.1093/cid/ciaa248/5803306 32161940 PMC7108125

[B47] Ramírez F. (1998). Glucocorticoids Induce a Th2 Response in Vitro. Dev. Immunol. 6 (3–4), 223–243. 10.1155/1998/73401 9814597 PMC2276017

[B48] Rocio L.-G. Utrero-Rico A. Talayero P. Lasa-Lazaro M. Ramirez-Fernandez A. Naranjo L.. (2020). Interleukin-6-Based Mortality Risk Model for Hospitalised COVID-19 Patients. J. Allergy Clin. Immunol. 146 (4): 799–807. 10.1016/j.jaci.2020.07.009 32710975 PMC7375283

[B49] Roncati L. Nasillo V. Lusenti B. Riva G. (2020). Signals of Th2 Immune Response from COVID-19 Patients Requiring Intensive Care. Ann. Hematol. 99 (6), 1419–1420. 10.1007/s00277-020-04066-7 32382776 PMC7205481

[B50] Rothan H. A. Byrareddy S. N. (2020). The Epidemiology and Pathogenesis of Coronavirus Disease (COVID-19) Outbreak. J. Autoimmun. 109, 102433 10.1016/j.jaut.2020.102433 PMC7127067 32113704

[B51] Sähr A. Förmer S. Hildebrand D. Heeg K. (2015). T-Cell Activation or Tolerization: The Yin and Yang of Bacterial Superantigens. Front. Microbiol. 6, 1153. 10.3389/fmicb.2015.01153 26539181 PMC4611159

[B52] Sakata-Kaneko S. Wakatsuki Y. Matsunaga Y. Usui T. Kita T. (2000). Altered Th1/Th2 Commitment in Human CD4+ T Cells with Ageing. Clin. Exp. Immunol. 120 (2), 267–273. 10.1046/j.1365-2249.2000.01224.x 10792375 PMC1905644

[B53] Schett G. Sticherling M. Neurath M. F. (2020). COVID-19: Risk for Cytokine Targeting in Chronic Inflammatory Diseases?” Nat. Rev. Immunol. 20 (5), 271–272. 10.1038/s41577-020-0312-7 32296135 PMC7186927

[B54] Song C. Y. Xu J. He J. Q. Lu Y. Q. (2020). Immune Dysfunction Following COVID-19, Especially in Severe Patients. Sci. Rep. 10 (1), 1–11. 10.1038/s41598-020-72718-9 32985562 PMC7522270

[B55] Spellberg B. Edwards J. E. (2001). Type 1/Type 2 Immunity in Infectious Diseases. Clin. Infect. Dis. 32 (1), 76–102. 10.1086/317537 11118387

[B56] Tan L. Wang Q. Zhang D. Ding J. Huang Q. Tang Y. Q.. (2020). Lymphopenia Predicts Disease Severity of COVID-19: A Descriptive and Predictive Study. Signal Transduct. Target. Ther. 52 (1), 33. 10.1038/s41392-020-0148-4 PMC7100419 32296069

[B57] Vabret N. Britton G. J. Gruber C. Hegde S. Kim J. Kuksin M.. (2020). Immunology of COVID-19: Current State of the Science. Immunity 52 (6), 910–941. 10.1016/j.immuni.2020.05.002 32505227 PMC7200337

[B58] Vardhana S. A. Wolchok J. D. (2020). The Many Faces of the Anti-COVID Immune Response. J. Exp. Med. 217 (6). 10.1084/JEM.20200678 PMC7191310 32353870

[B59] Vieira P. L. Kaliński P. Wierenga E. A. Kapsenberg M. L. de Jong E. C. (1998). Glucocorticoids Inhibit Bioactive IL-12p70 Production by In Vitro-Generated Human Dendritic Cells Without Affecting Their T Cell Stimulatory Potential. J. Immunol. 161, (10). 9820496

[B60] Wan Y. Y. (2010). Multi-Tasking of Helper T Cells. Immunology 130 (2), 166–171. 10.1111/j.1365-2567.2010.03289.x 20557575 PMC2878461

[B61] Wang W. K. Chen S. Y. Jung Liu I. Kao C. L. Chen H. L. Chiang B. L.. (2004). Temporal Relationship of Viral Load, Ribavirin, Interleukin (IL)-6, IL-8, and Clinical Progression in Patients with Severe Acute Respiratory Syndrome. Clin. Infect. Dis. 39 (7), 1071–1075. 10.1086/423808 15472864 PMC7107918

[B62] Wang F. Nie J. Wang H. Zhao Q. Xiong Y. Deng L.. (2020). Characteristics of Peripheral Lymphocyte Subset Alteration in COVID-19 Pneumonia. J. Infect. Dis. 221 (11), 1762–1769. 10.1093/infdis/jiaa150 32227123 PMC7184346

[B63] Wang F. Hou H. Luo Y. Tang G. Wu S. Huang M.. (2020). The Laboratory Tests and Host Immunity of COVID-19 Patients with Different Severity of Illness. JCI Insight 5 (10), e137799. 10.1172/jci.insight.137799 PMC7259533 32324595

[B64] Ward E. J. Fu H. Marelli-Berg F. (2017). Monitoring Migration of Activated T Cells to Antigen-Rich Non-Lymphoid Tissue.” In Methods Mol. Biol. 1591, 215–224. 10.1007/978-1-4939-6931-9_15 28349485

[B65] Wei L.-l. Wang W.-j. Chen D.-x. Xu B. (2020). Dysregulation of the Immune Response Affects the Outcome of Critical COVID-19 Patients. J. Med. Virol. 92 (11), 2768–2776 10.1002/jmv.26181 32543740 PMC7323247

[B66] Weiskopf D. Schmitz K. S. Raadsen M. P. Grifoni A. Okba N. M. A. Endeman H.. (2020). Phenotype and Kinetics of SARS-CoV-2-Specific T Cells in COVID-19 Patients with Acute Respiratory Distress Syndrome. Sci. Immunol. 5 (48), eabd2071. 10.1126/sciimmunol.abd2071 32591408 PMC7319493

[B67] WHO (2020). “Clinical Management of COVID-19 : interim guidance, 27 May 2020. World Health Organization. Document number: WHO/2019-nCoV/clinical/2020.5.” World Health Organization. Available from: https://apps.who.int/iris/handle/10665/332196. License: CC BY-NC-SA 3.0 IGO

[B68] Willheim M. Ebner C. Baier K. Kern W. Schrattbauer K. Thien R.. (1997). Cell Surface Characterization of T Lymphocytes and Allergen-Specific T Cell Clones: Correlation of CD26 Expression with TH1 Subsets. J. Allergy Clin. Immunol. 100 (3), 348–355. 10.1016/S0091-6749(97)70248-3 9314347

[B69] Wolff D. Nee S. Hickey N. S. Marschollek M. (2020). Risk Factors for Covid-19 Severity and Fatality: A Structured Literature Review. Infection 49 (1), 1 10.1007/s15010-020-01509-1 32860214 PMC7453858

[B70] Wong R. S. M. Wu A. To K. F. Lee N. Lam C. W. K. Wong C. K.. (2003). Haematological Manifestations in Patients with Severe Acute Respiratory Syndrome: Retrospective Analysis. Br. Med. J. 326 (7403), 1358–1362. 10.1136/bmj.326.7403.1358 12816821 PMC162124

[B71] Wong C. K. Lam C. W. K. Wu A. K. L. Ip W. K. Lee N. L. S. Chan I. H. S.. (2004). Plasma Inflammatory Cytokines and Chemokines in Severe Acute Respiratory Syndrome. Clin. Exp. Immunol. 136 (1), 95–103. 10.1111/j.1365-2249.2004.02415.x 15030519 PMC1808997

[B72] Wu D. Yang X. O. (2020). TH17 Responses in Cytokine Storm of COVID-19: An Emerging Target of JAK2 Inhibitor Fedratinib. J. Microbiol. Immunol. Infect. 53 (3), 368–370. 10.1016/j.jmii.2020.03.005 32205092 PMC7156211

[B73] Xu H. Zhong L. Deng J. Peng J. Dan H. Zeng X.. (2020). High Expression of ACE2 Receptor of 2019-NCoV on the Epithelial Cells of Oral Mucosa. Int. J. Oral. Sci. 12 (1), 1–5. 10.1038/s41368-020-0074-x 32094336 PMC7039956

[B74] Xu X. Han M. Li T. Sun W. Wang D. Fu B.. (2020). Effective Treatment of Severe COVID-19 Patients with Tocilizumab. Proc. Natl. Acad. Sci. United States America 117 (20), 10970–10975. 10.1073/pnas.2005615117 PMC7245089 32350134

[B75] Yang A. P. Liu J. p. Tao W. q. ming Li H. (2020). The Diagnostic and Predictive Role of NLR, d-NLR and PLR in COVID-19 Patients. Int. Immunopharmacol. 84 (July), 106504. 10.1016/j.intimp.2020.106504 32304994 PMC7152924

[B76] Yao X. H. Li T. Y. He Z. C. Ping Y. F. Liu H. W. Yu S. C.. (2020). [A Pathological Report of Three COVID-19 Cases by Minimally Invasive Autopsies]. Zhonghua Bing Li Xue Za Zhi = Chin. J. Pathol. 49 (0), E009. 10.3760/cma.j.cn112151-20200312-00193 32172546

[B77] Yuki K. Fujiogi M. Koutsogiannaki S. (2020). COVID-19 Pathophysiology: A Review. Clin. Immunol. 215 (June), 108427. 10.1016/j.clim.2020.108427 32325252 PMC7169933

[B78] Zhang Y. Li J. Zhan Y. Wu L. Yu X. Zhang W.. (2004). Analysis of Serum Cytokines in Patients with Severe Acute Respiratory Syndrome. Infect. Immun. 72 (8), 4410–4415. 10.1128/IAI.72.8.4410-4415.2004 15271897 PMC470699

[B79] Zheng H. Y. Zhang M. Yang C. X. Zhang N. Wang X. C. Yang X. P. (2020). Elevated Exhaustion Levels and Reduced Functional Diversity of T Cells in Peripheral Blood May Predict Severe Progression in COVID-19 Patients. Cell. Mol. Immunol. Springer Nat. 17 (5), 541–43. 10.1038/s41423-020-0401-3 PMC7091621 32203186

[B80] Zheng M. Gao Y. Wang G. Song G. Liu S. Sun D.. (2020). Functional Exhaustion of Antiviral Lymphocytes in COVID-19 Patients. Cell. Mol. Immunol. 17 (5), 533–535. 10.1038/s41423-020-0402-2 32203188 PMC7091858

[B81] Zheng Z. Peng F. Xu B. Zhao J. Liu H. Peng J.. (2020). Risk Factors of Critical & Mortal COVID-19 Cases: A Systematic Literature Review and Meta-Analysis. J. Infect. 81 (2), e16–e25. 10.1016/j.jinf.2020.04.021 PMC7177098 32335169

[B82] Zhou Y. Fu B. Zheng X. Wang D. Zhao C. qi Y.. Aberrant Pathogenic GM-CSF + T Cells and Inflammatory CD14 + CD16 + Monocytes 1 in Severe Pulmonary Syndrome Patients of a New Coronavirus 2 3. BioRxiv 10.1101/2020.02.12.945576

[B83] Zhou Y. Yang Z. Guo Y. Geng S. Gao S. Ye S.. (2020). A New Predictor of Disease Severity in Patients with COVID-19 in Wuhan, China” MedRxiv 10.1101/2020.03.24.20042119. March, 2020.03.24.20042119.

[B84] Zhu J. Yamane H. Paul W. E. (2010). Differentiation of Effector CD4 T Cell Populations” Annu. Rev. Immunol. 28 (1), 445–489. 10.1146/annurev-immunol-030409-101212 20192806 PMC3502616

