# Supplementary material for: T-Helper Cell Subset Response Is a Determining Factor in COVID-19 Progression
Source: Front Cell Infect Microbiol. 2021 Feb 26;11:624483. doi: 10.3389/fcimb.2021.624483 (PMC7952877; doi:10.3389/fcimb.2021.624483)
Supplement: Supplementary file 1 [file DataSheet_1.docx]

Supplementary Material

# Supplementary Data

Supplementary Material should be uploaded separately on submission. Please include any supplementary data, figures and/or tables. All supplementary files are deposited to FigShare for permanent storage and receive a DOI.

Supplementary material is not typeset so please ensure that all information is clearly presented, the appropriate caption is included in the file and not in the manuscript, and that the style conforms to the rest of the article. To avoid discrepancies between the published article and the supplementary material, please do not add the title, author list, affiliations or correspondence in the supplementary files.

# Supplementary Figures and Tables

For more information on Supplementary Material and for details on the different file types accepted, please see [here](http://home.frontiersin.org/about/author-guidelines#SupplementaryMaterial). Figures, tables, and images will be published under a Creative Commons CC-BY licence and permission must be obtained for use of copyrighted material from other sources (including re-published/adapted/modified/partial figures and images from the internet). It is the responsibility of the authors to acquire the licenses, to follow any citation instructions requested by third-party rights holders, and cover any supplementary charges.

## Supplementary Figures


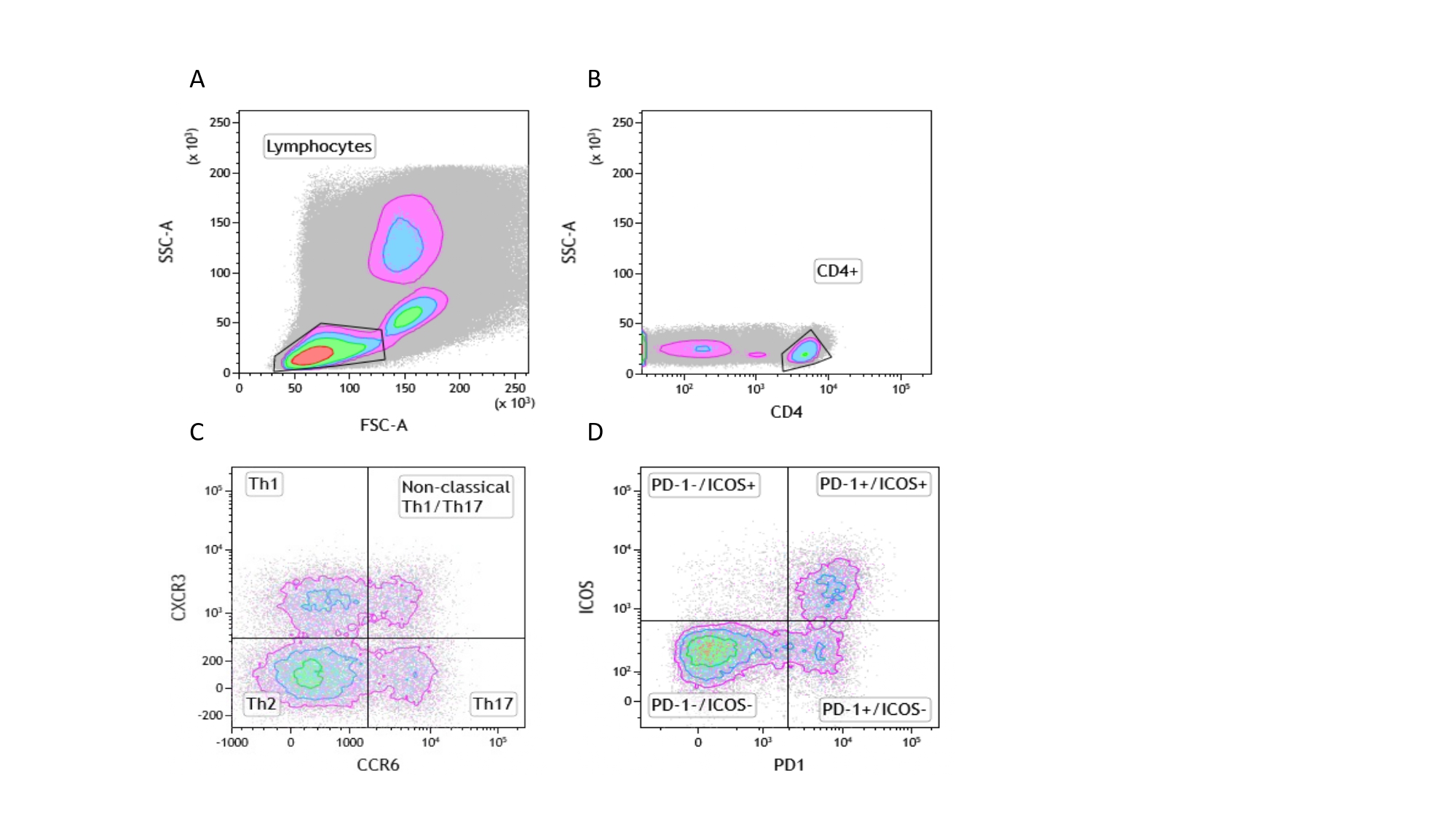


**Supplementary Figure 1.** Gaiting strategy of Th subsets. A) Lymphocytes B) T lymphocytes CD4+ from gated total lymphocytes C) CXCR3+/CCR6- (Th1). CXCR3-/CCR6- (Th2). CXCR3-/CCR6+ (Th17) from gated CD4 D) Activation (ICOS) or senescence (PD-1) from gated each Th subst.


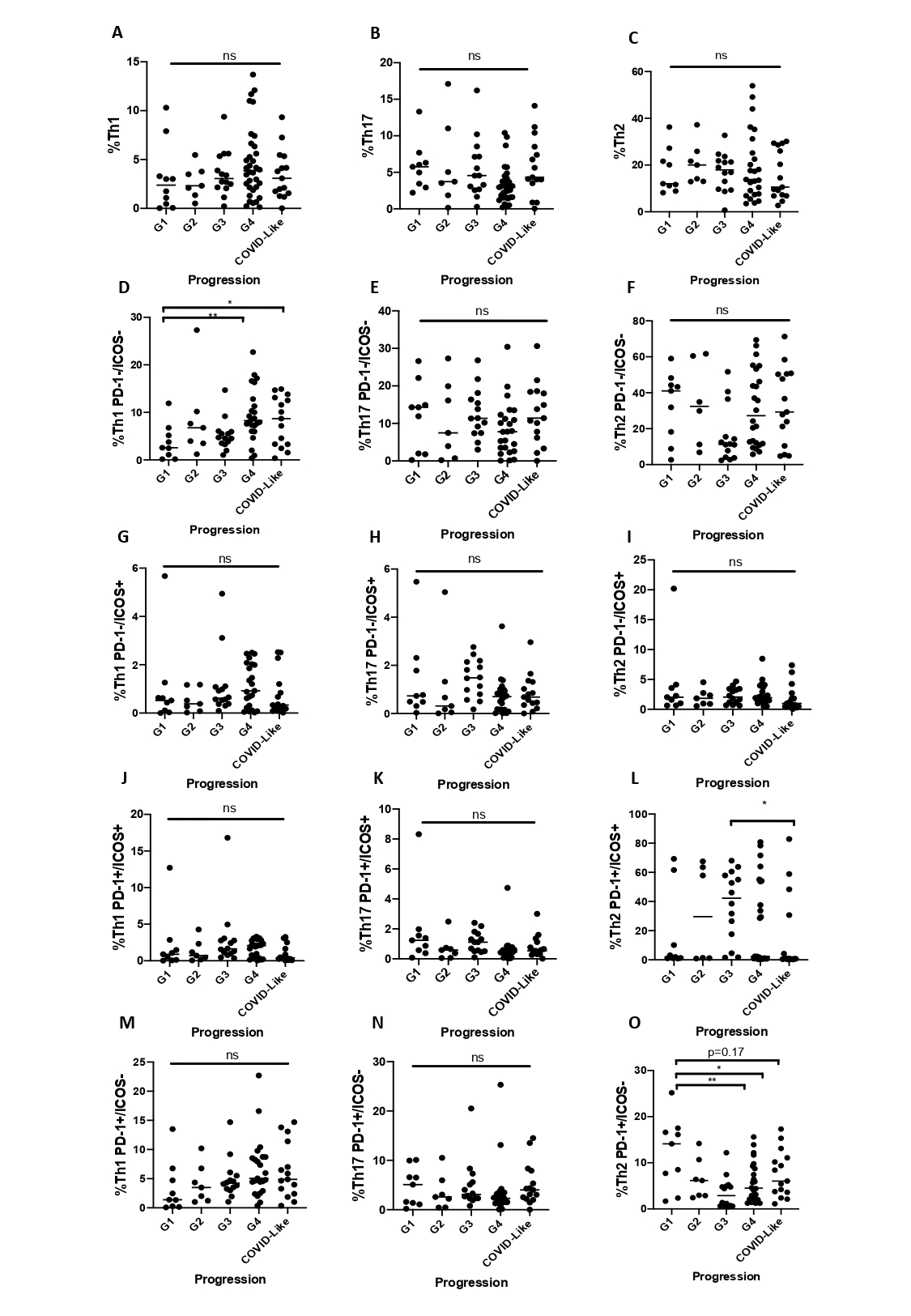


**Supplementary Figure 2.** Th distribution between COVID-19 patients according to the most critical event during disease and COVID-Like patients. A-C) Total proportion of Th 1 (A). Th17 (B) and Th2 (C). D-F) Proportion of quiescent Th1 (D), Th17 (E) and Th2 (F). G-I) Proportion of early activated Th1 (G), Th17 (H) and Th2 (I). J-L) Proportion of late activated Th1 (J), Th17 (K), Th2 (L). M-O) Proportion of senescent Th1 (M), Th17 (N) and Th2 (O).

G1: Death; G2: Intensive Care Unit; G3: Immunomodulators; G4: Benign-Course.

*. <0.05

**. <0.01

ns. not significant.

**
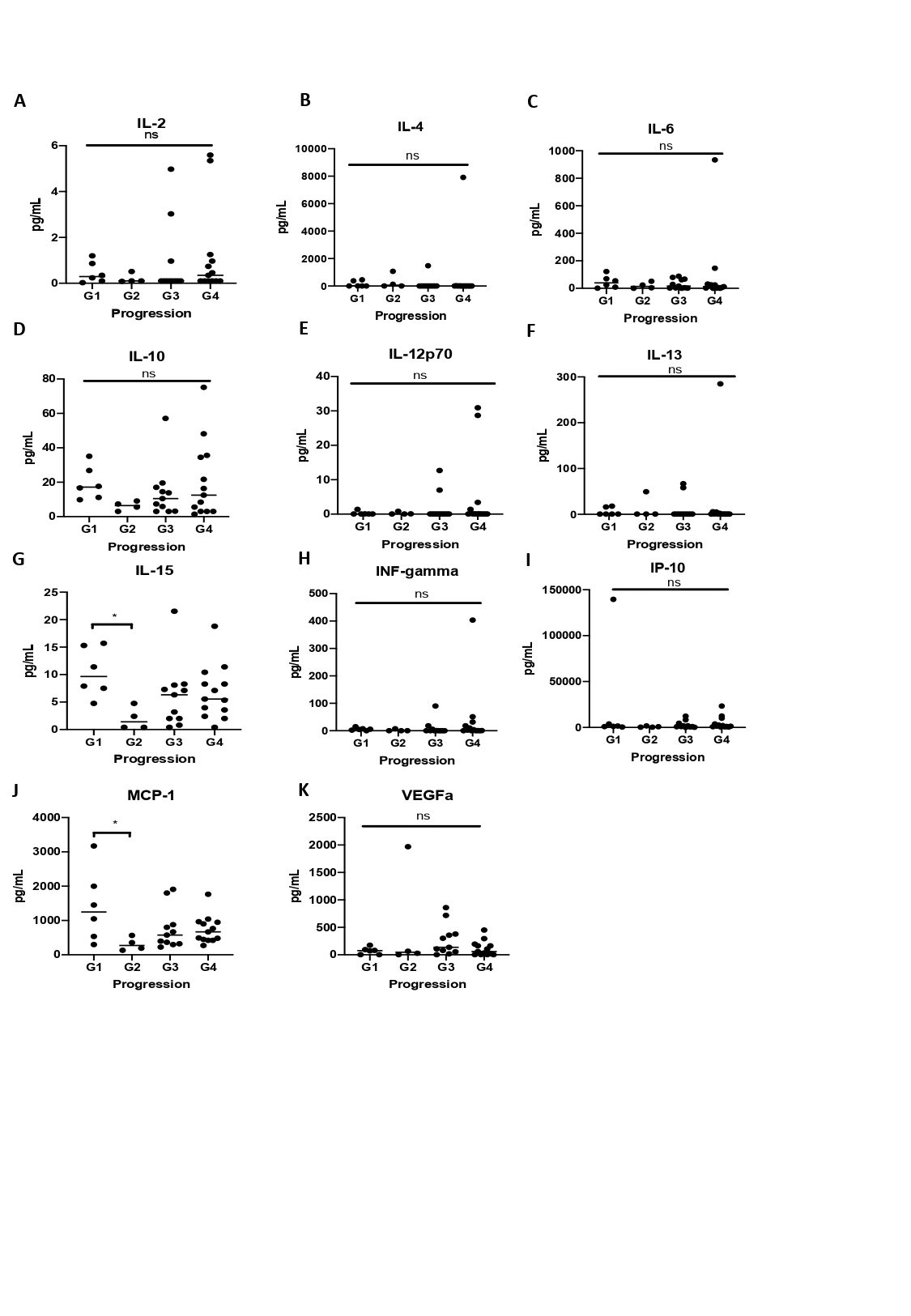
**

**Supplementary Figure 3.** Cytokine profile in COVID-19 patients according to the most critical event during disease. A) IL-2 B) IL4 C) IL-6 D) IL-10 E) IL12p70 F) IL-13 G) IL-15 H) INF-Gamma I) MCP-1 J) IP-10 K) VEGFa. G1: Death; G2: Intensive Care Unit; G3: Immunomodulators; G4: Benign-Course.

*.<0.05

ns. not significant

## Supplementary Tables

**Supplementary Table 1**. Comparison of the T helper cells between COVID-19 patients and reference population. PD-1-/ICOS-: Quiescent; PD-1-/ICOS+: Early-activated; PD-1+/ICOS+: Late-activated; PD-1+/ICOS: Senescent.

| **Th Cells %** | **Reference population N=21** | | **COVID-19 patients N=40** | | **p-value** |
| --- | --- | --- | --- | --- | --- |
|  | **Median** | **IQR** | **Median** | **IQR** |  |
| **%Th1** | 6.7 | 4.95-8.20 | 2.99 | 1.55-4.61 | **<0.001** |
| **%Th17** | 6.95 | 3.08-10.57 | 3.41 | 2.3-5.65 | **0.012** |
| **%Th2** | 16.8 | 11.37-20.90 | 17.1 | 10.02-23.1 | 0.88 |
| **%Th1 PD-1-/ICOS-** | 9.76 | 6.62-11.77 | 6 | 3.49-9.2 | **0.007** |
| **%Th17 PD-1-/ICOS-** | 13.2 | 5.25-18.35 | 10.1 | 3.65-15.27 | 0.19 |
| **%Th2 PD-1-/ICOS-** | 35.9 | 27.22-42.55 | 21.3 | 10.85-44.1 | 0.111 |
| **% Th1 PD-1-/ICOS+** | 1.17 | 0.70-2.06 | 0.65 | 0.3-1.26 | **0.026** |
| **%Th17 PD-1-/ICOS+** | 1.19 | 0.62-1.79 | 0.8 | 0.31-1.45 | 0.091 |
| **%Th2 PD-1-/ICOS+** | 2.81 | 1.35-3.60 | 2 | 1.19-3.42 | 0.368 |
| **%Th1 PD-1+/ICOS+** | 1.48 | 0.96-2.36 | 1.56 | 0.46-2.77 | 0.561 |
| **%Th17 PD-1+/ICOS+** | 1.44 | 0.57-2.10 | 0.58 | 0.39-1.2 | **0.007** |
| **%Th2 PD-1+/ICOS+** | 1.31 | 1.07-1.88 | 17.6 | 1.19-55.07 | **0.004** |
| **%Th1 PD-1+/ICOS-** | 4.27 | 3.03-7.65 | 4.55 | 2-7.78 | 0.78 |
| **%Th17 PD-1+/ICOS-** | 3.21 | 1.55-6.98 | 2.59 | 1.32-5.73 | 0.5 |
| **%Th2 PD-1+/ICOS-** | 4.52 | 3.1-6.49 | 4.7 | 2.12-9.15 | **<0.001** |

**Supplementary Table 2**. Comparison of T helper cells between COVID-19 and COVID-Like patients. PD-1-/ICOS-: Quiescent; PD-1-/ICOS+: Early-activated; PD-1+/ICOS+: Late-activated; PD-1+/ICOS: Senescent.

| **Th Cells %** | **COVID-Like patients N=15** | | **COVID-19 patients N=55** | | **p-value** |
| --- | --- | --- | --- | --- | --- |
|  | **Median** | **IQR** | **Median** | **IQR** |  |
| **%Th1** | 3.08 | 1.6-5.05 | 2.99 | 1.55-4.61 | 0.880 |
| **%Th17** | 4.33 | 6.63-8.2 | 3.41 | 2.3-5.65 | 0.146 |
| **%Th2** | 10.6 | 6.93-27.95 | 17.1 | 10.02-23.1 | 0.370 |
| **%Th1 PD-1-/ICOS-** | 8.6 | 3.29-12.95 | 6 | 3.49-9.2 | 0.494 |
| **%Th17 PD-1-/ICOS-** | 11.4 | 6.57-18.3 | 10.1 | 3.65-15.27 | 0.351 |
| **%Th2 PD-1-/ICOS-** | 29.3 | 13.1-50.45 | 21.3 | 10.85-44.1 | 0.458 |
| **% Th1 PD-1-/ICOS+** | 0.33 | 0.18-1.11 | 0.65 | 0.3-1.26 | 0.355 |
| **%Th17 PD-1-/ICOS+** | 0.68 | 0.37-1.28 | 0.8 | 0.31-1.45 | 0.667 |
| **%Th2 PD-1-/ICOS+** | 0.98 | 0.5-2.59 | 2 | 1.19-3.42 | 0.079 |
| **%Th1 PD-1+/ICOS+** | 0.4 | 0.26-2.28 | 1.56 | 0.46-2.77 | 0.145 |
| **%Th17 PD-1+/ICOS+** | 0.54 | 0.32-1.03 | 0.58 | 0.39-1.2 | 0.787 |
| **%Th2 PD-1+/ICOS+** | 0.76 | 0.44-24.08 | 17.6 | 1.19-55.07 | **0.006** |
| **%Th1 PD-1+/ICOS-** | 4.89 | 2.62-10.3 | 4.55 | 2-7.78 | 0.411 |
| **%Th17 PD-1+/ICOS-** | 4.02 | 2.27-7.26 | 2.59 | 1.32-5.73 | 0.195 |
| **%Th2 PD-1+/ICOS-** | 6.03 | 3.64-10.87 | 4.7 | 2.12-9.15 | 0.301 |

**Supplementary Table 3.** Cytokine profile comparison between COVID-19 patient and reference population.

| **Cytokines** | **Reference Population; N=94** | | **COVID-19 patients; N=34** | | **p-value** |
| --- | --- | --- | --- | --- | --- |
|  | **Median** | **IQR** | **Median** | **IQR** |  |
| **IL-2** | 0.1 | 0.1-0.1 | 0.1 | 0.1-0.76 | **< 0.001** |
| **IL-4** | 265.98 | 93.51-482.88 | 1.4 | 1.4-9.58 | **< 0.001** |
| **IL-6** | 0.08 | 0.07-5.15 | 19.72 | 2.26-54.54 | **< 0.001** |
| **IL-10** | 0.88 | 0.15-3.2 | 10.84 | 2.54-19.54 | **< 0.001** |
| **IL-12p70** | 2 | 2.01-2.02 | 0.08 | 0.05-0.08 | **< 0.001** |
| **IL-13** | 0 | 0-0.5 | 0.5 | 0.5-2.32 | **< 0.001** |
| **IL-15** | 0.09 | 0.09-0.99 | 5.93 | 2-8.28 | **< 0.001** |
| **INFγ** | 0.3 | 0.2-10.03 | 0.49 | 0.3-7.18 | **0.024** |
| **IP-10** | 331.24 | 203.69-471.24 | 1245.22 | 660.86-3775.03 | **< 0.001** |
| **MCP-1** | 643.23 | 487.62-834.42 | 569.74 | 364.22-966.39 | 0.742 |
| **VEGFα** | 100.99 | 72.69-127-66 | 80.72 | 17.45-193.94 | 0.676 |

**Supplementary Table 4.** Categorization of Th subsets based on Gaussian distribution

| **Variables** | **Low** | **Medium** | **High** |
| --- | --- | --- | --- |
| **Total lymphocytes** | <850 | 850-1200 | >1200 |
| **%Th1** | <3 | 3-8 | >8 |
| **%Th17** | <6 | - | >6 |
| **%Th2** | <17.5 | 17.5-32.5 | >32.5 |
| **%Th1 PD-1-/ICOS-** | <5 | 5-13 | >13 |
| **%Th17 PD-1-/ICOS-** | <10 | - | >10 |
| **%Th2 PD-1-/ICOS-** | <21 | - | >21 |
| **% Th1 PD-1-/ICOS+** | <1.2 | - | >1.2 |
| **%Th17 PD-1-/ICOS+** | <0.9 | - | >0.9 |
| **%Th2 PD-1-/ICOS+** | <3.5 | 3.5-8 | >8 |
| **%Th1 PD-1+/ICOS+** | <4 | - | >4 |
| **%Th17 PD-1+/ICOS+** | <2.5 | - | >2.5 |
| **%Th2 PD-1+/ICOS+** | <24 | - | >24 |
| **%Th1 PD-1+/ICOS-** | <9 | - | >9 |
| **%Th17 PD-1+/ICOS-** | <5 | - | >5 |
| **%Th2 PD-1+/ICOS-** | <6 | - | >6 |
